# Comparison of case-based and lecture-based learning in dental fluorosis diagnostic ability with visual analog scale assessment

**DOI:** 10.1186/s12909-024-05695-6

**Published:** 2024-07-15

**Authors:** Man Wang, Shanshan Liang, Tao Jiang

**Affiliations:** 1 Department of Stomatology, Union Hospital, Tongji Medical College, Huazhong University of Science and Technology, Wuhan, 430022 China; 2 School of Stomatology, Tongji Medical College, Huazhong University of Science and Technology, Wuhan, 430030 China; 3 Hubei Province Key Laboratory of Oral and Maxillofacial Development and Regeneration, Wuhan, 430022 China; 4 State Key Laboratory of Oral & Maxillofacial Reconstruction and Regeneration, Key Laboratory of Oral Biomedicine Ministry of Education, Hubei Key Laboratory of Stomatology, School & Hospital of Stomatology, Wuhan University, 237 Luoyu Road, Wuhan, 430079 China; 5 Department of Prosthodontics, Hospital of Stomatology, Wuhan University, 237 Luoyu Road, Wuhan, 430079 China

**Keywords:** Case-based learning, Dental fluorosis, VAS, Reliability, Validity

## Abstract

**Objective:**

This study aimed to compare the impact of case-based learning (CBL) versus lecture-based learning (LBL) on dental students' clinical decision-making regarding DF severity using Visual Analog Scale (VAS) scoring.

**Methods:**

Eighty first-year graduate dental students were randomly assigned to either the CBL (*n* = 38) or LBL (*n* = 42) groups. Both groups received instruction on DF diagnosis, with CBL involving small group sessions analyzing real cases and LBL involving traditional lectures. Effectiveness was assessed by presenting 32 dental fluorosis cases with Thylstrup-Fejerskov Index (TSIF) scores ranging from 0 to 7 through slide presentations to both groups for VAS assessment. Five evaluators of each group randomly selected were asked to repeat the rating 2 weeks later. Statistical analysis included two-way ANOVA for group and gender differences, intra-class correlation coefficient (ICC) for reliability, and Spearman correlation coefficients for validity.

**Results:**

Variations in VAS scores were observed between CBL and LBL groups, with no significant gender impact. Excellent inter- and intra-evaluator agreement was found for VAS scoring in both groups, indicating its reliability. Validation against established indices (such as DI and TSIF) demonstrated strong correlations, with CBL students exhibiting higher correlations.

**Conclusions:**

CBL enhances students' clinical decision-making and proficiency in DF diagnosis, as evidenced by more consistent and accurate VAS scoring compared to LBL. These findings highlight the importance of innovative educational strategies in dental curricula, with implications for improving training quality and clinical outcomes.

**Trial registration:** The study was registered at the Clinical Research Center, Hospital of Stomatology, Wuhan University (Registration code: HGGC-036).

## Introduction

Fluorosis, a condition defined by dental fluorosis (DF), constitutes a prevalent public health concern that markedly affects dental esthetics and functionality [1]. The ingestion of fluoride culminates in dental brown staining, enamel opacity, and potential dental decay [2]. Precise determination and grading of DF severity are essential for effective therapeutic interventions and management protocols. Currently, several indices including the Dean Index(DI), Thylstrup and Fejerskov Index (TFI), Tooth Surface Index of Fluorosis (TSIF) and Visual Analog Scale (VAS) are widely employed for DF diagnosis [3]. Thereinto, the VAS serves as a commonly used tool for quantifying DF visibility, yielding a numeric score originally developed for pain measurement [4, 5]. The VAS offers rapid completion, generates ratio data (that is, a linear scale), exhibits high sensitivity to change, is easy to score and has good construct validity [6].

Numerous studies have employed VAS scales to assess the frequency and intensity of different symptoms in epidemiological and clinical research, such as the manifestation of DF [7–9]. However, despite its advantages, the diagnostic and evaluative utilization of VAS scoring in DF encounters complexities, particularly in clinical applications [10]. Predominantly, the subjective nature of VAS scoring may engender discrepancies among student practitioners in interpreting DF visibility, potentially leading to inconsistent therapeutic approaches. For example, a study conducted by Worthington et al. found that when clinicians were asked to assess the esthetic changes in implant dentistry using VAS, there was notable variation in their ratings [11]. So does Baccetti’s research on the assessment of smile esthetics by orthodontists [12]. Furthermore, the absence of standardized operational guidelines for VAS implementation in dental clinic further complicates its use. Student practitioners may lack adequate training in VAS usage, precipitating errors in assessment. Zechner’s research revealed that VAS did not show any good correlation to objective esthetic indices proposed so far[13]. The intricacies associated with VAS scoring in diagnosing DF in clinical contexts highlight the need for a more effective educational approach.

Traditional teaching methods in dental education, such as lecture-based learning (LBL) primarily focuses on knowledge transmission, often inadequately preparing students for real-world clinical scenarios [14]. LBL leads to passive understanding, lacks hands-on practice needed for clinical skills, emphasizes rote memorization over critical thinking, does not accommodate diverse learning paces, and provides insufficient immediate feedback, hindering students' ability to gauge and improve their understanding [15, 16]. In contrast, case-based learning (CBL) engages students actively with real clinical cases, bridging the gap between theory and practice. CBL enhances critical thinking and problem-solving skills, accommodates diverse learning styles, and provides immediate feedback, thus better preparing students for clinical decision-making [17–19]. Hence, CBL offers more structured guidance and clear learning objectives, ensuring a more focused and efficient learning experience. This makes it a compelling alternative for students to tackle diverse levels of DF cases, nurturing a nuanced comprehension of diagnostic challenges and assessment intricacies.

Therefore, it is hypothesized that students immersed in CBL will exhibit superior clinical acumen in diagnosing DF using VAS scoring compared to their LBL counterparts. This expectation stems from the notion that CBL, through its immersive approach with real cases and immediate feedback, cultivates a more nuanced understanding of DF assessment nuances, benefits for effective therapeutic interventions further. To verify the hypothesis, this study aimed to compare CBL with LBL in terms of its impact on dental students’ clinical decision making on DF severity by using the VAS scoring. Additionally, their reliability and validity of VAS in both groups were evaluated. The findings from this study will inform dental education reforms and contribute to enhancing the quality of dental training, particularly in the context of CBL implementation.

## Methods

### Participants

The study protocol was approved by the medical ethics committee of School & Hospital of Stomatology, Wuhan University. Conducted during the academic year 2022–23, the study involved first-year graduate dental students at the School of Stomatology, Wuhan University, who did not exhibit color blindness or other cognitive impairment. A total of 80 participants were selected and randomly assigned into two groups: the experimental group, taught with CBL (n = 38) and the control group, taught with LBL (*n* = 42). There was no statistical difference between the two groups in terms of gender, age, entrance achievement, self-study ability and subject preference (*p* > 0.05). Table 1 illustrates the general characteristics of the 80 participants.

**Table 1:** Distribution of number, gender, and age (Mean ± Standard Deviation) of two groups of participants

	CBL group		LBL group	
Gender	Number	Age (Mean ± Standard Deviation)	Number	Age (Mean ± Standard Deviation)
Male	15	23.86 ± 0.74	17	23.41 ± 1.06
Female	23	23.61 ± 1.12	25	23.40 ± 0.76

### Design

In the LBL group, students were received instruction through traditional lectures held in a classroom setting, covering a variety of topics related to DF. This course aimed to provide comprehensive understanding of the etiology, pathophysiology, clinical manifestations, diagnosis, operational guidelines of various evaluative methods for DF severity, with particular emphasis on VAS. The traditional learning sessions lasted $45 min per course, with a total of 4 courses delivering the entirety of the teaching content.

Students in CBL group participated in small group sessions, each consisting of seven or eight students. Prior to these sessions, the instructor, with the assistance of physicians from the affiliated hospital, selected typical DF patients exhibiting varying severity and obtained their permission for disease history inquiries and clinical examination by students. Students were encouraged to engage in self-directed learning of DF-related information. During the teaching sessions, students conducted oral clinical examinations, analyzed each case, assessed severity using VAS and discussed factors contributing to the severity. Each session included five patients with varying degrees of DF, ensuring an even distribution of TSIF values among them, with each group of CBL students taking turns to examine three to four of them. At the end, instructors summarized and evaluated students' discussion outcomes, highlighted key diagnostic points, addressed any teaching-related issues encountered, and solicited feedback from students for course evaluation. Similar to the control group, teaching sessions in the experimental group also lasted 45 min per course, totaling 4 courses. Different patients were invited for each of the four sessions, provided with compensation for their time and reimbursement for travel expenses, supported by the fund.

The inclusion criteria of typical DF patients were as follows:

- Age 19–38
- Presence of two fluorosed maxillary central incisors
- No carious or non-carious lesion in these two teeth
- No filling or prosthesis in these two teeth
- No periodontal inflammation
- Acceptance to be taken frontal view photography of incisors

Exclusion criteria included:

- Poor general or dental health
- Pregnant or lactating
- Smoking habit
- Use of any fixed orthodontic appliances
- Previous use of bleaching agents

### Effectiveness assessment

The effectiveness and satisfaction of the two teaching methods were evaluated by presenting the identical DF cases to students in both groups to assess their clinical decision making. Each evaluator received a written informed instruction detailing the study's purpose and procedures and signed a consent form to participate in the study.

#### DF cases selection and photography

DF cases were selected following a simple random sampling procedure from patients visiting the department of prosthodontics, Hospital of Stomatology, Wuhan University, between January 1st, 2022 and December 30th, 2023. The maxillary incisors and canines of these patients were evaluated and diagnosed as DF by prosthodontics specialist according to inclusion criteria and. exclusion criteria. Each patient signed an informed consent form after receiving an explanation of the study's aims and procedures at the study's outset.

**Fig. 1:**
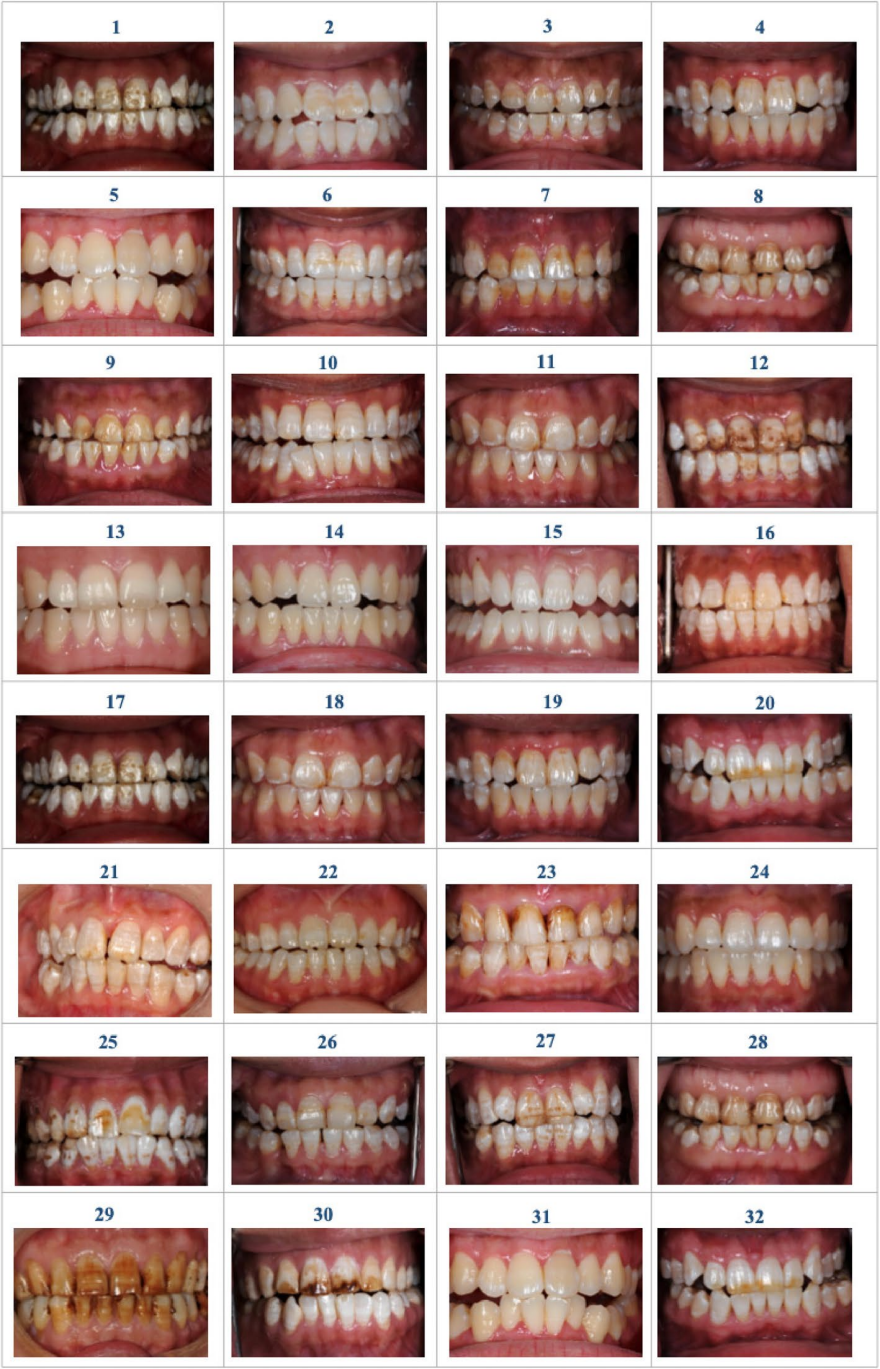
32 images of DF cases with TSIF scores ranging from 0 to 7

All intraoral photographs were taken using a Nikon D700 camera and Nikon 105 mm-macro lens, with consistent lighting conditions. The magnification was set at 1:2, with an aperture of F25, a shutter speed of 1/125, and flash intensity of M/4. To expose the incisors, canines, and premolars, patients' lips were held open with a mouth gag, and only one photographer captured the images to ensure consistency. The photographs were subsequently transferred to a personal computer-based system for storage and viewing. Photoshop software was utilized to crop each image to focus on a single maxillary central tooth (wide 3.4 mm and height 3.8 mm). Totally, 105 images were obtained and evaluated by two prosthodontics experienced in DF assessment, following the grading standard of TSIF. From these images, 32 teeth with TSIF scores ranging from 0 to 7 were randomly selected using random number tables and arranged for further analysis (Fig. 1).

#### Evaluation

All the students in both groups rate the severity of each case presented through a slideshow, with each case displayed for ten seconds. Using a 100 mm horizontal VAS, students placed a vertical mark to indicate their assessment of each case. The left end of the scale represented the best or ideal tooth, while the right end represented the worst or unacceptable tooth. The photographs were shown in random order to each evaluator to minimize bias.

We measured the distance between the left end and the marked point made by each student for every photograph. Two weeks later, five students were randomly selected from each group and then rated the cases once again.

### Data analysis

Statistical analyses were conducted using SPSS 28.0 (SPSS, Chicago, IL, USA) for WINDOWS. The results of VAS scores were presented as mean ± SD. Two-way ANOVA were analyzed differences between groups and gender for all 32 cases. *P* < 0.05 was considered statistically significant.

#### Reliability

Reliability, in the context of this study, refers to the consistency of scores over time, between different evaluators, or across different tasks or items measuring the same construct. It is typically assessed using a reliability coefficient, which measures the proportion of variance in scores that is related to the true variance between the objects being measured. In this study, reliability, including both inter-examiner agreement and intra-examiner agreement, was assessed using the intra-class correlation coefficient (ICC) [20, 21].

**Inter-evaluator reliability** refers to the consistency of ratings among different evaluators. The ICC is utilized to measure the level of agreement among evaluators for each group. In this study, both "single ICC" and "mean ICC" were calculated by two-way random model in SPSS. Single ICC represents the reliability for a single evaluator, while mean ICC represents the reliability for multiple evaluators. Furthermore, ICC was employed to examine whether any differences existed in how the CBL group perceived DF compared to the LBL group. [22].

**Intra-evaluator reliability** assesses the consistency of ratings made by the same evaluator over time. Five evaluators from each group were asked to repeat the test and rate the cases again. ICC was used to evaluate the intra-evaluator reliability for each of these five evaluators. Pearson correlation coefficients (R) were applied to assess the correlation of each evaluator's repeated evaluations, with benchmarks provided by Landis and Koch (< 0.4 poor agreement; 0.4–0.6 moderate agreement; 0.6–0.8 substantial agreement; > 0.8 excellent agreement) [23, 24].

#### Validation

Validity refers to the accuracy and appropriateness of a measurement tool in assessing the intended construct [25]. The Spearman rank correlation coefficient (R_S_) is often used to assess the validity of a measurement by examining the strength and direction of the relationship between its scores and those of a criterion measure [26]. Higher R_S_ indicate stronger validity, suggesting that the measurement tool reliably reflects the underlying construct [25, 27].

**Validation of mean scores:** Spearman rank correlation coefficients between the mean VAS score of each case in relation to DI and TSIF of that case;

**Validation of total scores:** Spearman rank correlation coefficients between the total VAS scores of each case assessed by all evaluators in relation to DI and TSIF of that case;

**Validation of each evaluator:** Spearman rank correlation coefficients between the VAS scores of each case assessed by each evaluator in relation to DI and TSIF of that case.

## Results

### Group and gender differences

**Fig. 2:**
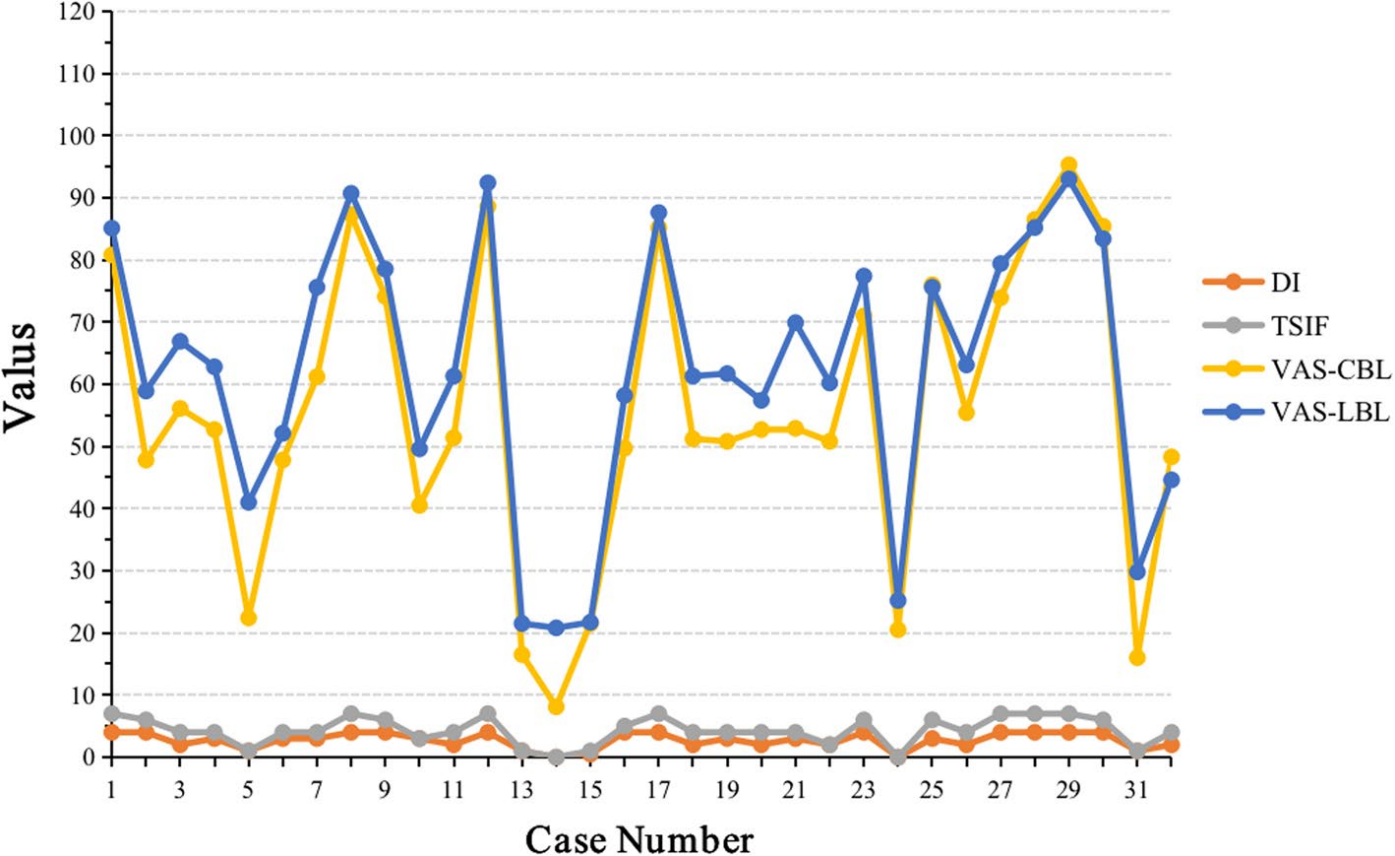
The VAS scores Distribution in CBL and LBL Groups for 32 DF cases

The VAS, DI and TSIF scores (Mean ± SD) for 32 DF cases were listed in Table 2 and Fig. 2. In the CBL group, VAS scores ranged from 8.1 ± 7.79 to 95.3 ± 5.03, while in the LBL group, they ranged from 20.8 ± 14.73 to 93.0 ± 7.42. Significant differences were observed between the two groups for 14 cases, while no significant differences were observed for the remaining cases (Table 2, Fig. 2 and Table 3). Two-way ANOVA analysis of variance showed that neither the gender factor nor the interaction factor between group and gender showed a statistically significant impact on VAS.

**Table 2:** The VAS scores (Mean ± SD) of LBL and CBL groups for 32 DF cases

No	DI	TSIF	VAS		No	DI	TSIF	VAS	
			CBL group	LBL group				CBL group	CBL group
1	4	7	80.8 ± 9.53	85.1 ± 11.50	17	4	7	85.2 ± 10.69	87.6 ± 8.68
2*	4	6	47.8 ± 19.70	58.9 ± 16.89	18*	2	4	51.2 ± 16.44	61.3 ± 16.37
3*	2	4	56.1 ± 17.73	66.9 ± 13.24	19*	3	4	50.8 ± 15.91	61.7 ± 15.36
4*	3	4	52.7 ± 17.47	62.8 ± 16.24	20	2	4	52.7 ± 15.15	57.4 ± 16.80
5*	1	1	22.4 ± 14.95	41.0 ± 19.39	21*	3	4	52.9 ± 16.37	69.9 ± 13.66
6	3	4	47.8 ± 17.48	52.1 ± 16.53	22*	2	2	50.8 ± 15.36	60.2 ± 16.15
7*	3	4	61.2 ± 16.75	75.6 ± 13.53	23	4	6	71.0 ± 14.66	77.4 ± 12.01
8	4	7	87.3 ± 10.41	90.7 ± 8.99	24	0	0	20.5 ± 13.55	25.2 ± 16.08
9	4	6	74.1 ± 14.06	78.5 ± 13.86	25	3	6	76.0 ± 13.01	75.6 ± 14.12
10*	3	3	40.5 ± 19.34	49.6 ± 18.41	26*	2	4	55.4 ± 17.91	63.1 ± 18.07
11*	2	4	51.4 ± 17.68	61.3 ± 14.00	27	4	7	73.9 ± 14.86	79.4 ± 15.41
12	4	7	88.6 ± 15.59	92.4 ± 7.31	28	4	7	86.5 ± 7.81	85.2 ± 12.39
13	1	1	16.5 ± 12.93	21.5 ± 17.73	29	4	7	95.3 ± 5.03	93.0 ± 7.42
14*	0	0	8.1 ± 7.79	20.8 ± 14.73	30	4	6	85.4 ± 11.18	83.4 ± 13.22
15	0.5	1	21.5 ± 14.77	21.7 ± 14.90	31*	1	1	16.0 ± 12.24	29.8 ± 21.86
16	4	5	49.7 ± 19.00	58.2 ± 18.39	32	2	4	48.3 ± 18.89	44.6 ± 21.59

^*^ indicates statistically significant differences in VAS scores between groups

**Table 3:** The p-value of the two-way ANOVA analysis of VAS for Group and Gender factors of 32 cases

Case Number	1	2*	3*	4*	5*	6	7*	8
Group	0.107	0.009	0.006	0.011	< 0.001	0.177	< 0.001	0.186
Gender	0.636	0.722	0.204	0.113	0.113	0.704	0.074	0.748
Group* Gender	0.563	0.831	0.248	0.793	0.793	0.202	0.672	0.323
Case Number	9	10*	11*	12	13	14*	15	16
Group	0.266	0.035	0.013	0.272	0.104	< 0.001	0.891	0.076
Gender	0.849	0.187	0.72	0.316	0.496	0.426	0.340	0.413
Group* Gender	0.221	0.763	0.394	0.315	0.239	0.510	0.663	0.331
Case Number	17	18*	19*	20	21*	22*	23	24
Group	0.417	0.004	0.003	0.253	< 0.001	0.012	0.057	0.174
Gender	0.263	0.947	0.882	0.425	0.585	0.695	0.889	0.111
Group* Gender	0.215	0.16	0.692	0.569	0.199	0.972	0.483	0.978
Case Number	25	26*	27	28	29	30	31*	32
Group	0.942	0.048	0.124	0.778	0.132	0.651	< 0.001	0.613
Gender	0.219	0.226	0.877	0.857	0.509	0.600	0.814	0.268
Group* Gender	0.871	0.492	0.907	0.218	0.879	0.226	0.156	0.102

Group (CBL group and LBL group; Gender (male and female); Group*Gender (interaction factor between group and gender. Cases marked with * (2*, 3*, 4*, 5*, 7*, 10*, 11*, 14*, 18*, 19*, 21*, 22*, 26*, 31*) represents significant difference in VAS between the CBL and LBL group. Neither the gender factor nor the interaction factor between group and gender showed a statistically significant impact on VAS

### Reliability

Inter-evaluator reliability, as indicated by the ICC, demonstrated excellent agreement among evaluators in both two groups. **(**Table 4**)** For single evaluation, total ICC was 0.719 (*p* < 0.001) in CBL group, and 0.685 (*p* < 0.001) in LBL group. For mean evaluation, total ICC was 0.990 (*p* < 0.001) in CBL group, and 0.989 (*p* < 0.001) in LBL group.

**Table 4:** The single ICC and mean ICC values of LBL and CBL groups

		CBL group			LBL group		
		Male	Female	Total	Male	Female	Total
ICC	Single evaluation	0.701	0.723	0.719	0.661	0.685	0.685
	P value	< 0.001	< 0.001	< 0.001	< 0.001	< 0.001	< 0.001
ICC	Mean evaluation	0.972	0.984	0.990	0.971	0.982	0.989
	P value	< 0.001	< 0.001	< 0.001	< 0.001	< 0.001	< 0.001

The ICC is calculated by two-way random model, representing absolute agreement

Intra-evaluator reliability, assessed by Pearson correlation coefficients of twice measurements, ranged from 0.894 to 0.940. Single ICC values ranged from 0.621–0.928 and mean ICC values ranged from 0.766 to 0.962. **(**Table 5**)**. According to Landis and Koch’s, the ICC showed good or excellent intra-evaluator correlations.

**Table 5:** The Pearson correlation coefficients, single ICC and mean ICC values of LBL and CBL groups

	Evaluator	Pearson correlation coefficients	Single ICC	Mean ICC
CBL group	1	0.940^**^	0.914^**^	0.955^**^
	2	0.898^**^	0.808^**^	0.894^**^
	3	0.935^**^	0.911^**^	0.953^**^
	4	0.894^**^	0.888^**^	0.941^**^
	5	0.896^**^	0.621^**^	0.766^**^
LBL group	1	0.937^**^	0.928^**^	0.962^**^
	2	0.926^**^	0.899^**^	0.947^**^
	3	0.805^**^	0.701^**^	0.824^**^
	4	0.873^**^	0.865^**^	0.927^**^
	5	0.901^**^	0.892^**^	0.943^**^

^**^ indicates *P* < 0.001

### Validation

#### Validation of mean and total VAS scores for DF against DI and TSIF

Spearman correlation coefficients of VAS (mean) versus DI, TSIF were 0.762 and 0.882 (*p* < 0.001) for CBL group, and 0.818, 0.900 (*P* < 0.001) for LBL group, respectively **(**Table 6**)**. The R_S_ of VAS (total) versus DI, TSIF were 0.723 and 0.898 (*p* < 0.001) for CBL group, and 0.679, 0.741 (*P* < 0.001) for LBL group, respectively.

**Table 6:** The Spearman correlation coefficients (Rs) of VAS (mean scores and total scores) versus DI, TSIF in LBL and CBL groups

	CBL group		LBL group	
	Mean VAS	Total VAS	Mean VAS	Total VAS
DI	0.762^**^	0.723^**^	0.818^**^	0.679^**^
TSIF	0.882^**^	0.798^**^	0.900^**^	0.741^**^

^**^ indicates *P* < 0.001

#### Validation of VAS of each evaluator against DI and TSIF

**Fig. 3:**
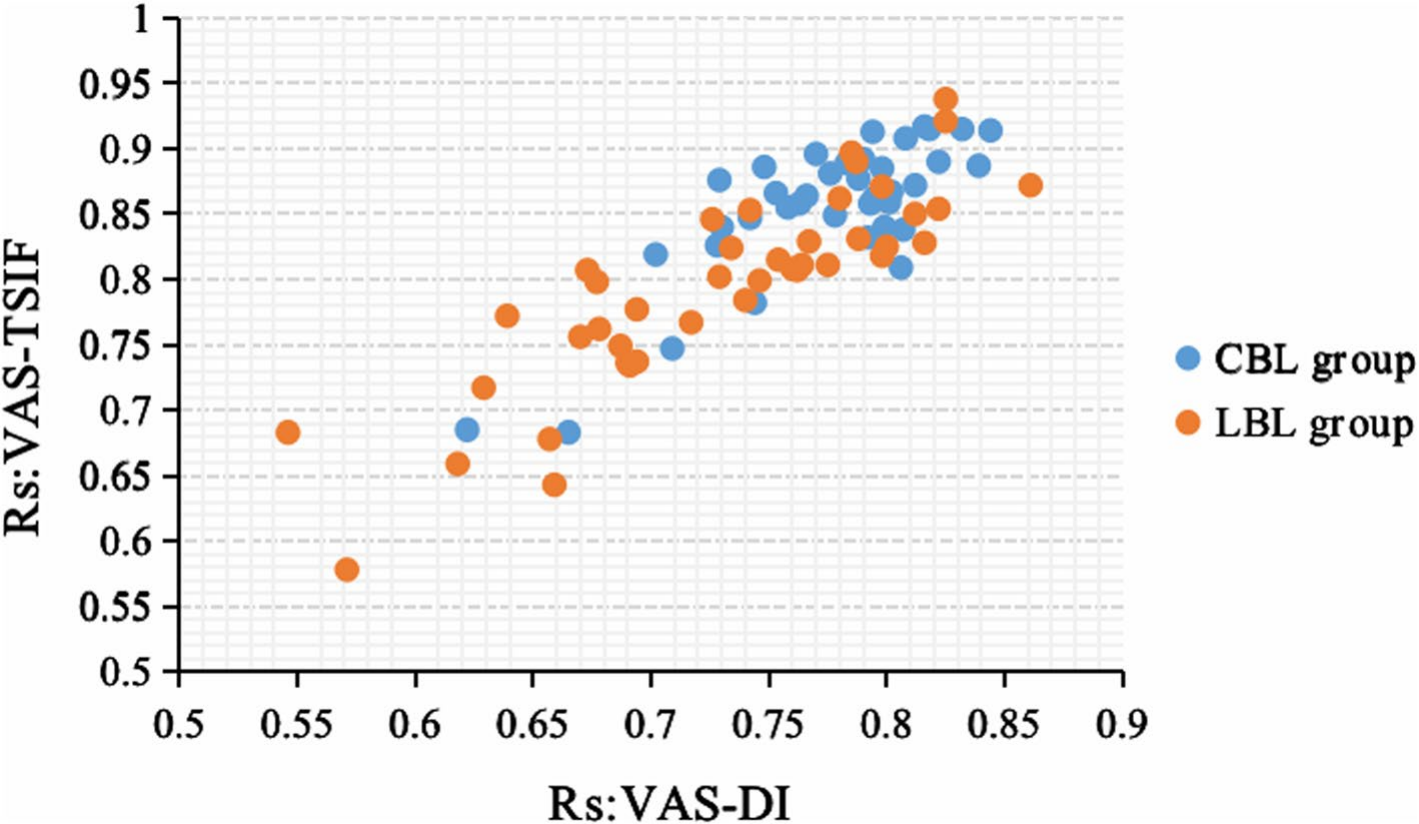
The Rs: VAS-DI vs. Rs: VAS-TSIF Scatter Plot for CBL and LBL Groups

For Rs between VAS with TSIF: Excellent correlations (Rs > 0.8) have been found in both two groups including 34 evaluators in CBL group and 24 ones in LBL group. In the CBL group, 4 evaluators were considered to have good correlation (0.6 < RS < 0.8), while in the LBL group, 17 evaluators had good correlation and one evaluator had moderate correlation (0.4 < RS < 0.6) (Table 7 and Fig. 3).

**Table 7:** The spearman correlation coefficients (Rs) of VAS score of each case assessed by each evaluator versus DI, TSIF in LBL and CBL groups

CBL group						LBL group					
No	Rs:VAS-DI	Rs:VAS-TSIF	No	Rs:VAS-DI	Rs:VAS-TSIF	No	Rs:VAS-DI	Rs:VAS-TSIF	No	Rs:VAS-DI	Rs:VAS-TSIF
1	0.807^**^	0.838^**^	20	0.816^**^	0.917^**^	1	0.787^**^	0.890^**^	22	0.678^**^	0.762^**^
2	0.753^**^	0.866^**^	21	0.832^**^	0.915^**^	2	0.780^**^	0.862^**^	23	0.734^**^	0.824^**^
3	0.778^**^	0.849^**^	22	0.729^**^	0.876^**^	3	0.659^**^	0.643^**^	24	0.775^**^	0.811^**^
4	0.794^**^	0.913^**^	23	0.766^**^	0.864^**^	4	0.726^**^	0.846^**^	25	0.677^**^	0.798^**^
5	0.844^**^	0.914^**^	24	0.806^**^	0.809^**^	5	0.764^**^	0.811^**^	26	0.546^**^	0.683^**^
6	0.748^**^	0.886^**^	25	0.622^**^	0.685^**^	6	0.785^**^	0.897^**^	27	0.717^**^	0.767^**^
7	0.770^**^	0.896^**^	26	0.742^**^	0.847^**^	7	0.861^**^	0.872^**^	28	0.762^**^	0.807^**^
8	0.799^**^	0.840^**^	27	0.798^**^	0.885^**^	8	0.673^**^	0.807^**^	29	0.760^**^	0.808^**^
9	0.665^**^	0.683^**^	28	0.818^**^	0.915^**^	9	0.670^**^	0.756^**^	30	0.812^**^	0.850^**^
10	0.790^**^	0.892^**^	29	0.776^**^	0.881^**^	10	0.571^**^	0.578^**^	31	0.798^**^	0.818^**^
11	0.702^**^	0.819^**^	30	0.709^**^	0.747^**^	11	0.639^**^	0.772^**^	32	0.788^**^	0.831^**^
12	0.758^**^	0.855^**^	31	0.730^**^	0.840^**^	12	0.694^**^	0.777^**^	33	0.816^**^	0.828^**^
13	0.763^**^	0.858^**^	32	0.792^**^	0.832^**^	13	0.729^**^	0.802^**^	34	0.690^**^	0.736^**^
14	0.801^**^	0.859^**^	33	0.783^**^	0.889^**^	14	0.740^**^	0.784^**^	35	0.767^**^	0.829^**^
15	0.744^**^	0.782^**^	34	0.728^**^	0.826^**^	15	0.691^**^	0.734^**^	36	0.746^**^	0.799^**^
16	0.822^**^	0.890^**^	35	0.788^**^	0.877^**^	16	0.657^**^	0.678^**^	37	0.800^**^	0.825^**^
17	0.802^**^	0.867^**^	36	0.812^**^	0.872^**^	17	0.687^**^	0.749^**^	38	0.754^**^	0.815^**^
18	0.793^**^	0.858^**^	37	0.839^**^	0.887^**^	18	0.825^**^	0.938^**^	39	0.798^**^	0.871^**^
19	0.795^**^	0.863^**^	38	0.808^**^	0.908^**^	19	0.742^**^	0.853^**^	40	0.618^**^	0.659^**^
						20	0.694^**^	0.737^**^	41	0.629^**^	0.717^**^
						21	0.825^**^	0.921^**^	42	0.822^**^	0.854^**^

** indicates *P* < 0.001

Similar trends were observed for the correlation between VAS and DI: Excellent correlations (RS > 0.8) were found in both groups, with 12 evaluators in the CBL group and 6 evaluators in the LBL group. In the CBL group, 26 evaluators were considered to have good correlation, while in the LBL group, 34 evaluators had good correlation and two evaluators had moderate correlation (0.4 < RS < 0.6) (Table 7 and Fig. 3).

## Discussion

DF poses a significant public health concern due to its impact on both the appearance and functionality of dentition. Cases of fluorosis, characterized by brown staining and enamel opacity, underscore the need of early detection and intervention, so accurate diagnosis and grading of DF severity are paramount for effective therapeutic strategies. Guiding dental students in DF diagnosis and assessment is vital within educational curricula. This study compared the effectiveness of CBL and LBL in improving dental students' clinical decision-making regarding DF using VAS scoring. Unlike LBL, which primarily focuses on conveying knowledge through lectures, CBL emphasizes real clinical cases, providing students with a practical understanding of DF diagnosis and VAS scoring.

Analysis of VAS scores among the CBL and LBL groups revealed variations in certain DF cases. Gender did not significantly impact VAS scores, suggesting comparable performance between male and female students in assessing DF severity. The reliability of VAS was assessed through inter- and intra-evaluator agreement, indicating excellent agreement among evaluators in both groups. High ICC values for single and mean evaluations within each group underscored the method's reliability in assessing DF severity.

The validation of VAS scores against established indices like DI and TSIF provides insights into its accuracy. Strong correlation coefficients between VAS scores and DI/TSIF values indicate its reliability, with consistent correlations across evaluators enhancing its robustness. Importantly, Spearman correlation coefficients between total VAS scores and DI/TSIF were consistently higher in the CBL group compared to LBL group, suggesting a more accurate understanding of DF severity among CBL-exposed students. Furthermore, the correlation analysis for each evaluator unveils a similar trend. In both the CBL and LBL groups, the majority of evaluators demonstrate excellent correlations (Rs > 0.8) between VAS scores and TSIF/DI. However, it's noteworthy that the CBL group consistently exhibits a higher proportion of excellent correlations, with fewer evaluators showing moderate correlations (0.4 < Rs < 0.6) compared to the LBL group. This indicates that students exposed to CBL may develop a more consistent and accurate approach to DF assessment using VAS scoring. For analyzing the observed differences between two groups, several potential confounding variables should also be considered. The teaching quality and style may affect students’ response to teaching methods and VAS scoring [28]. Additionally, differences in time spent on course material, individual learning preferences for CBL or LBL can influence proficiency [29]. Furthermore, student motivation and engagement [30], along with potential assessment biases [31], should also be considered to ensure fair evaluation.

The study preliminarily confirmed significant performance advantages among students in the experimental group, attributed to the effectiveness of CBL. Firstly, through case-based learning and discussion, students are exposed to a diverse DF cases, empowering them to formulate correct clinical diagnostic and examination descriptions with emphasis and standardization [32]. Secondly, the CBL method, utilizing teacher-set questions, facilitates students in analyzing DF cases, enabling them to make a clear diagnosis based on symptoms and preliminary oral examination results, thereby augmenting their clinical reasoning abilities [33]. Thirdly, each case includes questions pertaining to the severity grading of dental fluorosis, supplemented by relevant clinical videos, guiding students to consider diagnostic standards and techniques [34]. Engaging in independent literature review and video analysis, students deepen their understanding and impression of clinical diagnosis and treatment, indirectly enhancing proficiency and accuracy [34].

Additionally, the integration of CBL into dental education could address broader educational objectives. Firstly, CBL cultivates critical thinking by immersing students in real clinical cases, prompting active engagement and analysis. It prompts students to apply theoretical knowledge to practical scenarios, encouraging problem-solving and preparing them for the complexities of dental practice [35]. Secondly, CBL encourages interdisciplinary collaboration by prompting students to consider diverse perspectives and expertise when approaching clinical cases. This collaboration, exemplified in scenarios such as DF diagnosis, enhances students' appreciation for teamwork and communication skills vital for effective patient care [36]. Moreover, CBL nurtures lifelong learning by fostering a curiosity-driven approach to education. Through exposure to diverse cases and supplementary resources, students are empowered to explore new concepts, conduct independent research, and continually update their skills, crucial in dentistry's dynamic landscape of evolving research and technology [37].

Integrating CBL into dental education for DF diagnosis using VAS scoring presents a promising approach to address these educational objectives. However, several potential barriers must be addressed to ensure effective delivery. Resource constraints, such as limited access to diverse clinical cases and insufficient technological support, may hinder the implementation of CBL. Faculty training in CBL design and facilitation is also crucial to ensure the quality and consistency of instruction. Additionally, innovative strategies are needed to enhance student engagement and motivation, particularly in virtual or hybrid learning environments. Addressing these potential barriers would provide a more balanced perspective on the educational approach.

The associated costs and value must also be considered [38]. While CBL offers a rich learning experience through real clinical cases and active student engagement, its resource-intensive nature, especially in dentistry with limited access to diverse cases and technology, is a concern. The expenses for CBL come from the need for clinical resources, faculty training, and technology. Future research should include comparative cost–benefit analyses against other methods to make informed decisions on adopting and optimizing CBL in dental education, ensuring sustainability and scalability.

Acknowledging the study's limitations, such as its reliance on a relatively small sample size and its focus on a single educational institution, is also crucial. Future research endeavors could explore the long-term impact of CBL on students' clinical competencies and evaluate the generalizability of these findings across diverse dental education settings. Moreover, delving deeper into the factors influencing VAS scoring discrepancies among student practitioners, and the cost–benefit analyses could pave the way for developing standardized guidelines for its clinical application.

## Conclusion

In conclusion, this study examined the efficacy of CBL versus LBL in improving dental students' proficiency in grading DF severity using VAS scoring. The findings indicate that students exposed to CBL exhibited a superior comprehension of DF severity, as evidenced by notable discrepancies in VAS scores. This underscores the significance of adopting innovative educational methodologies to better equip future dental practitioners for clinical practice. Moving forward, future research endeavors should involve collaborative efforts across multiple institutions, incorporating larger sample sizes and longitudinal observation periods. Such initiatives are crucial for bolstering the study's reliability and applicability across diverse educational contexts.

## Data Availability

The data that support the findings of this study are not openly available due to reasons of sensitivity and are available from the corresponding author upon reasonable request. Data are located in controlled access data storage at Wuhan University.
